# Pharmacogenetic study of the impact of ABCB1 single-nucleotide polymorphisms on lenalidomide treatment outcomes in patients with multiple myeloma: results from a phase IV observational study and subsequent phase II clinical trial

**DOI:** 10.1007/s00280-017-3481-8

**Published:** 2017-11-25

**Authors:** Ingrid Jakobsen Falk, Johan Lund, Henrik Gréen, Astrid Gruber, Evren Alici, Birgitta Lauri, Cecilie Blimark, Ulf-Henrik Mellqvist, Agneta Swedin, Karin Forsberg, Conny Carlsson, Mats Hardling, Lucia Ahlberg, Kourosh Lotfi, Hareth Nahi

**Affiliations:** 1 Division of Drug Research, Department of Medical and Health Sciences, Linköping University, Linköping, Sweden; 2 Unit for Hematology, Department of Medicine, Karolinska Institute, Huddinge, Sweden; 3 Department of Forensic Genetics and Forensic Toxicology, National Board of Forensic Medicine, Linköping, Sweden; 4 Department of Internal Medicine, Sunderby Hospital, Luleå, Sweden; 5 Hematology Department, Sahlgrenska University Hospital, Gothenburg, Sweden; 6 Division for Haematology, Oncology and Lung, Department of Medicine, South Elvsborg Hospital, Borås, Sweden; 7 Hematology Department, Skåne University Hospital, Lund, Sweden; 8 Department of Hematology, Norrland University Hospital, Umeå, Sweden; 9 Department of Internal Medicine, Hallands Hospital, Halmstad, Sweden; 10 Department of Hematology, Uddevalla Hospital, Uddevalla, Sweden; 11 Department of Hematology, Linköping University Hospital, Linköping, Sweden

**Keywords:** Lenalidomide, Multiple myeloma, Genetic markers, Single-nucleotide polymorphisms, P-Glycoprotein

## Abstract

**Purpose:**

Despite therapeutic advances, patients with multiple myeloma (MM) continue to experience disease relapse and treatment resistance. The gene *ABCB1* encodes the drug transporter P-glycoprotein, which confers resistance through drug extrusion across the cell membrane. Lenalidomide (Len) is excreted mainly via the kidneys, and, given the expression of P-gp in the renal tubuli, single-nucleotide polymorphisms (SNPs) in the *ABCB1* gene may influence Len plasma concentrations and, subsequently, the outcome of treatment. We, therefore, investigated the influence of *ABCB1* genetic variants on Len treatment outcomes and adverse events (AEs).

**Methods:**

Ninety patients with relapsed or refractory MM, who received the second-line Len plus dexamethasone in the Rev II trial, were genotyped for the *ABCB1* SNPs 1199G>A (Ser400Asn, rs2229109), 1236C>T (silent, rs1128503), 2677G>T/A (Ala893Ser, rs2032582), and 3435C>T (silent, rs1045642) using pyrosequencing, and correlations to response parameters, outcomes, and AEs were investigated.

**Results:**

No significant associations were found between genotype and either best response rates or hematological AEs, and 1236C>T, 2677G>T or 3435C>T genotypes had no impact on survival. There was a trend towards increased time to progression (TTP) in patients carrying the 1199A variant, and a significant difference in TTP between genotypes in patients with standard-risk cytogenetics.

**Conclusions:**

Our findings show a limited influence of *ABCB1* genotype on lenalidomide treatment efficacy and safety. The results suggest that 1199G>A may be a marker of TTP following Len treatment in standard-risk patients; however, larger studies are needed to validate and clarify the relationship.

**Supplementary Material:**

Supplementary data to this article can be found online at 10.1007/s00280-017-3481-8.

## Introduction

Considerable progress has been made in the treatment of multiple myeloma (MM) over the past 2 decades. Overall survival (OS) has more than doubled for some patients since the introduction of the proteasome inhibitor bortezomib and the immunomodulatory drugs (IMiDs) thalidomide and lenalidomide (Len) [1, 2]. The mode of action of IMiDs has been under investigation since the introduction of thalidomide in the 1960s, but has only recently been described in detail [3]. Agents of this class bind to cereblon, an ubiquitin ligase complex that, in turn, ubiquitinates IKZF1 and IKZF3. These two proteins are important for the upregulation of Myc and IRF4, two further proteins that are essential to the MM cell. Ubiquitination of IKZF1 and IKZF3 leads to their degradation via the proteasome, and downregulation of Myc and IRF4.

The gene *ABCB1* encodes the drug transporter P-glycoprotein (P-gp), which is responsible for the extrusion of a wide variety of drugs across the cell membrane. This is a known resistance mechanism in cancer [4]. In addition to upregulation of P-gp, single-nucleotide polymorphisms (SNPs) affecting transporter expression and activity may influence the plasma drug concentrations—and, subsequently, the response to treatment—of known P-gp substrates.

The influence of *ABCB1* SNPs on outcomes in MM has been investigated with various treatment regimens and in different patient cohorts [5]. The results are inconclusive, with some studies reporting conflicting correlations for the SNPs studied, and others finding no significant impact on outcomes [6–10].

Len, which is approved for the treatment of relapsed or refractory MM (RRMM) largely based on the results of two phase III clinical trials [11, 12], undergoes limited metabolism, and is mainly excreted via the kidneys [13]. In vitro studies have shown Len to be a P-gp substrate and, given the expression of P-gp at the brush border of renal tubular cells, SNPs in the *ABCB1* gene may influence Len plasma concentrations and, thus, the outcome of Len treatment [14]. The most extensively studied variants are SNPs 1236C>T (silent, rs1128503), 2677G>T/A (Ala893Ser, rs2032582) and 3435C>T (silent, rs1045642), but the 1199G>A (Ser400Asn, rs2229109) variant has also been reported to have functional implications [15, 16].

We investigated the four above-mentioned *ABCB1* SNPs in MM patients enrolled in the Rev II clinical trial of second-line Len-based treatment [17], and the impact of these polymorphisms on treatment response, hematological adverse events (AEs), and survival.

## Materials and methods

Rev II was a prospective, multicenter clinical trial that, between 2010 and 2013, enrolled 133 patients who had received one previous line of treatment for RRMM. The study was performed in two parts. In the first, observational part (ClinicalTrials.gov identifier NCT01430546), Len-naïve patients experiencing first relapse were treated with Len plus dexamethasone (Len + Dex) according to standard local clinical practice for ≤ 9 cycles of 4 weeks’ duration. In the second part, patients who achieved at least a partial response (PR) in the observational part of the study response was determined according to International Myeloma Working Group (IMWG) uniform response criteria [18], and then received ≥ 2 additional cycles of Len + Dex as consolidation treatment, were invited to participate in a prospective, randomized, open-label, multicenter, interventional, phase II clinical trial (NCT01450215). Sixty-two patients entered the phase II trial and were randomized (1:1) to either continuous Len + Dex or single-agent Len for ≤ 24 cycles or until disease progression or unacceptable toxicity. All patients consented to participation, and the study was performed in accordance with the ethical principles of the Helsinki Declaration.

For the present SNP analysis, samples were available for genotyping from 90 patients enrolled in the observational part of the Rev II trial. Of these patients, 47 were further randomized in the interventional phase II study: 23 to Len + Dex and 24 to single-agent Len. DNA was isolated using the Promega Maxwell 16 system (Promega Biotech AB, Sweden), and genotyping of the *ABCB1* SNPs 1199G>A (Ser400Asn, rs2229109), 1236C>T (silent, rs1128503), 2677G>T/A (Ala893Ser, rs2032582), and 3435C>T (silent, rs1045642) was carried out by pyrosequencing on a PyroMark 96 MD instrument (Qiagen, Sweden) according to the manufacturer’s instructions. Briefly, polymerase chain reactions (PCRs), with biotinylation of one primer in each primer pair, were performed in total reaction volumes of 10 μl. HotStar Taq PCR Mastermix (VWR, Sweden) was used for the reactions, with a magnesium chloride concentration of 1.5 mM and a final primer concentration of 0.4 μM. The annealing temperature was 58 °C, and the PCR was run for 50 cycles. Single-stranded biotinylated DNA templates were then prepared, and sequencing primers were annealed to the templates for 2 min at 80 °C. Enzyme and substrate were added, and the sequencing reactions were performed by adding nucleotides in a predefined dispensing order. Primer sequences and dispensing orders are presented in supplemental Table SI.

### Statistical analyses

Time to progression (TTP), time to next treatment (TTNT, defined as time between the start of the current line of treatment and start of the next line of treatment; physicians’ choice) and OS were assessed using Kaplan–Meier analyses with the log-rank test for significance. Cox regression (forced entry method) was used for multivariable survival analyses, adjusting for age, gender, hemoglobin, creatinine, albumin, fluorescence in situ hybridization (FISH) high-risk cytogenetics [presence of del17p13, add 1q21, and/or t(4;14)], previous treatment, Eastern Cooperative Oncology Group (ECOG) performance status at inclusion, International Staging System (ISS) disease stage at diagnosis, and previous high-dose therapy plus stem cell transplantation (HDT-SCT). Patients known to be alive at the end of the study were censored in the survival analysis at the date of the last follow-up. A *P* value of < 0.05 was considered significant, and a *P* value of 0.05–0.1 was considered a trend in the survival analyses.

Analyses were performed using all available material from the observational study, as well as from subgroups of patients defined by the presence/absence of high-risk cytogenetic features. Patients randomized in the subsequent phase II trial were also analyzed separately and according to treatment received. In analyses of the impact of genotype on response, patients were grouped as achieving ≥ PR or < PR, and as achieving either at least or less than a very good partial response (≥ VGPR or < VGPR). Time to response (measured from the date of inclusion to the date of first response/date of best response) and duration of response (DoR; time from first response to progression or death, with censoring at the date of last follow-up) were also assessed. Frequencies of hematological AEs (anemia, neutropenia, and thrombocytopenia) were compared between genotype groups using the Chi-square test; frequencies of grade 1, 2, 3, and 4 AEs were compared separately, and frequencies of grade 1–2 and grade 3–4 AEs were also compared. Distributions of patient baseline characteristics were compared between genotype groups using Mann–Whitney *U* or Kruskal–Wallis tests for continuous variables, and Chi-square or generalized Fisher´s exact tests for categorical variables. A *P* value of < 0.05 was considered significant. Median follow-up times were compared between genotype groups using an independent samples median test.

## Results

### Genotyping and patient characteristics

Baseline demographics and disease characteristics for the 90 patients included in this analysis are summarized in Table 1. Genotyping for the four *ABCB1* SNPs 1199G>A (Ser400Asn, rs2229109), 1236C>T (silent, rs1128503), 2677G>T/A (Ala893Ser, rs2032582), and 3435C>T (silent, rs1045642) was successfully performed for all patients (Table 2). Genotype frequencies did not differ significantly from the frequencies reported for a Nordic reference population genotyped with the same method [19]. The low-frequency 2677A allele was excluded from the analysis.

**Table 1:** Patient characteristics

	Total *n* = 90
Mean age, years (range)	67 (42–86)
Gender, n (%)	
Male	49 (45.6)
Female	41 (54.4)
Mean white blood cell count, × 109/l (range)	5.4 (1.4–14)
Mean hemoglobin, g/l (range)	115 (63–155)
Mean creatinine, μmol/l (range)	81.6 (42–270)
Mean albumin, g/l (range)	35 (25–44)
Presence of cytogenetic aberrations, n/N tested (%)	
8p21 deletion	16/75 (21.3)
13q deletion	25/76 (32.9)
p53 deletion	8/75 (10.7)
t(4;14)	5/42 (11.9)
t(11;14)	13/40 (32.5)
High-risk by FISH	35/75 (46.7)
M-component	
Class, n (%)	
IgA	18 (20)
IgG	60 (66.7)
IgM	1 (1.1)
Bence Jones	11 (12.2)
Light chain, n/N tested (%)	*N* = 88
Kappa	55 (62.5)
Lambda	33 (37.5)
Previous bone disease	68 (75.6)
ISS stage at diagnosis, n/N tested (%)	*N* = 67
Stage 1	16 (23.9)
Stage 2	41 (61.2)
Stage 3	10 (14.9)
ECOG performance status at inclusion, n/N tested (%)	*N* = 85
0	39 (45.9)
1	39 (45.9)
2	7 (8.2)
Previous treatment, n (%)	
Velcade	51 (56.7)
Thalidomide	10 (11.1)
PI + IMiD	6 (6.7)
Other	23 (25.6)
Previous HDT-SCT, n (%)	50 (55.6)
First response, n (%)	
CR	1 (1.1)
nCR	4 (4.4)
VGPR	5 (5.6)
PR	67 (74.4)
Minimal/no response	11 (12.2)
Progression	2 (2.2)
≥PR	77 (85.6)
≥VGPR	10 (11.1)
Best response, n (%)	
CR	12 (13.3)
nCR	13 (14.4)
VGPR	17 (18.9)
PR	35 (38.9)
Minimal/no response	11 (12.2)
Progression	2 (2.2)
≥PR	77 (85.6)
≥VGPR	42 (46.7)
Status, n (%)	
Progressed	53 (58.9)
Alive at last follow-up	55 (61.1)
Deceased	35 (38.9)

*CR* complete response, *ECOG* Eastern Cooperative Oncology Group, *FISH* fluorescence in situ hybridization, *HDT-SCT* high-dose therapy plus stem cell transplantation, *Ig* immunoglobulin, *IMiD* immunomodulatory drug, *ISS* International Staging System, *nCR* near complete response, *PI* proteasome inhibitor, *PR* partial response, *VGPR* very good partial response

**Table 2:** Genotype frequencies (*N* = 90) for 1199G>A (Ser400Asn, rs2229109), 1236C>T (silent, rs1128503), and 3435C>T (silent, rs1045642)

*ABCB1* SNP	*n* (%)
1199G>A (Ser400Asn, rs2229109)	
G/G	76 (84.4)
G/A	14 (15.6)
1236C>T (silent, rs1128503)	
C/C	38 (42.2)
C/T	39 (43.3)
T/T	13 (14.4)
2677G>T/A (Ala893Ser, rs2032582)	
G/G	35 (38.9)
G/T	39 (43.3)
T/T	13 (14.4)
G/A	2 (2.2)
T/A	1 (1.1)
3435C>T (silent, rs1045642)	
C/C	18 (20.0)
C/T	49 (54.4)
T/T	23 (25.6)

*SNP* single-nucleotide polymorphism

At inclusion, there were no differences between genotypes in terms of hemoglobin, white blood cell, creatinine or albumin levels, or proportions of male versus female patients, previous treatment regimens, cytogenetic abnormalities, M-component disease subtype, ISS disease stage, or performance status. There were significant differences in the distribution of patient ages between genotypes for 1236C>T (*P* = 0.018), 2677G>T (*P* = 0.008), and 3435C>T (*P* = 0.046), and also significant differences in the rate of previous HDT-SCT (*P* = 0.028, *P* = 0.002, and *P* = 0.024 for 1236C>T, 2677G>T, and 3435C>T, respectively). This was reflected also in the results for patients with the TTT haplotype (carrying at least one T-allele in all three positions 1236, 2677, and 3435) being younger and more often subject to previous HDT-SCT compared to patients with other haplotypes; *P* = 0.009 for age and *P* = 0.002 for HDT-SCT. There was no difference in age distribution or other patient characteristics for the 1199G>A SNP. A significant difference between genotypes was seen in the rate of previous bone disease for the 2677G>T SNP (*P* = 0.049). For details on differences in demographics between genotypes, see supplemental Table SII.

### Efficacy outcomes

Median length of follow-up for the entire population was 3.0 years (range 0.2–5.3 years); with no significant differences between genotype groups.

Among all 90 patients included in these analyses, the response rate (≥ PR) was 85.6%, including 46.7% ≥ VGPR (Table 1). Time to first response (≥ PR) was 60 days (95% confidence interval [CI] 50–71), and time to best response (≥ PR) was 158 days (95% CI 120–195). Mean TTP was 2.4 years (95% CI 2.0–2.8), mean TTNT was 2.2 years (95% CI 1.9–2.6), and mean OS was 3.7 years (95% CI 3.2–4.1).

**Fig. 1:**
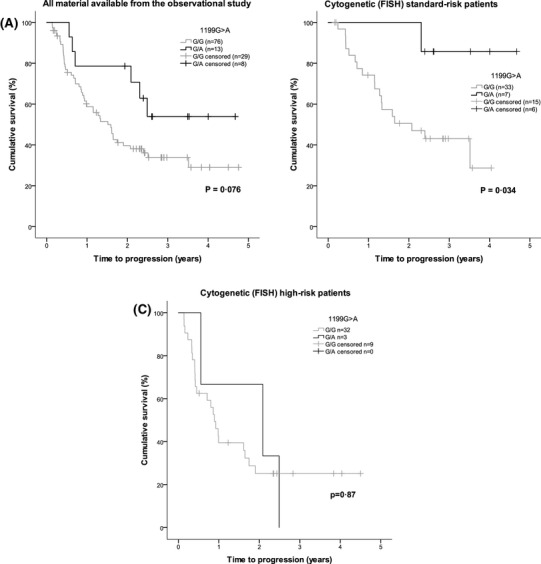
Kaplan–Meier analysis of time to progression (TTP) in relation to *ABCB1* SNP 1199G>A genotype, with log-rank test for significance. There was a trend towards prolonged TTP in patients with the heterozygous G/A genotype versus those carrying the G/G genotype; mean TTP was 3.2 years (95% CI 2.3–4.1) versus 2.2 years (95% CI 1.8–2.6), respectively; *P* = 0.076 (**a**). The potential influence of 1199G>A genotype appeared to be confined mainly to patients with standard-risk cytogenetics (**b**). Mean TTP was 2.3 years (95% CI 1.8–2.8) versus 4.3 years (95% CI 3.7–4.9) for standard-risk patients with the G/G versus the G/A genotype, *P* = 0.034. No significant difference was seen in the high-risk group (**c**). Mean TTP was 1.7 years (1.1–2.3 95% CI) versus 1.7 years (0.56–2.9 95% CI) for high-risk patients with the G/G versus the G/A genotype, *P* = 0.87. *CI* confidence interval, *FISH* fluorescence in situ hybridisation, *SNP* single-nucleotide polymorphism

**Fig. 2:**
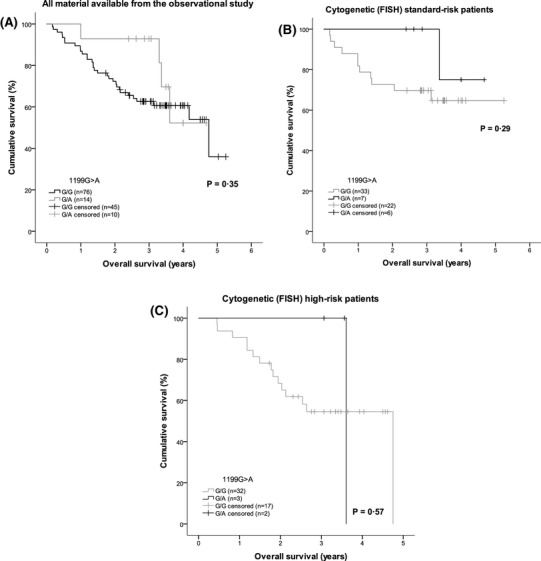
Kaplan–Meier analysis of overall survival (OS) in relation to the *ABCB1* SNP 1199G>A genotypes, with log-rank test for significance. OS results showed similar patterns to TTP (Fig. 1) in the overall patient population (*N* = 90) (**a**), in the standard-risk patient subgroup (**b**) and in the high-risk patient subgroup (**c**), although without any statistically significant differences. *FISH* fluorescence in situ hybridisation, *SNP* single-nucleotide polymorphism, *TTP* time to progression

Patients with 2677T/T and 3435T/T genotype appeared to have a higher rate of first response ≥ VGPR (*P* = 0.037 and *P* = 0.04, respectively). However, the number of patients with ≥ VGPR as their first response was low, and patients with these genotypes were also younger and more often subjected to the previous HDT-SCT (Table SII). In addition, the association to first response was not seen in the portion of the patients randomized in the second, interventional part, when analyzed separately (*n* = 47, *P* > 0.05, data not shown). There were no significant associations between any of the four *ABCB1* SNPs and best response to treatment; response rates by genotype are presented in supplemental Table SIII. There were no significant correlations with time to first response or best response for any of the polymorphisms. No influence on survival was seen in relation to genotype for the 1236C>T, 2677G>T, and 3435C>T SNPs. A trend was seen for longer TTP among patients carrying the 1199A allele—heterozygous G/A versus homozygous G/G patients (Fig. 1a, *P* = 0.076). This trend towards an association was also seen in the multivariable Cox regression analysis, adjusting for age, gender, hemoglobin, creatinine, albumin, high-risk cytogenetics, previous treatment, performance status at inclusion, ISS disease stage at diagnosis, and previous HDT-SCT (hazard ratio 0.280; 95% CI 0.74–1.054; *P* = 0.06). Other factors significantly associated with TTP in the Cox regression analysis were albumin, high-risk cytogenetics, and previous HDT-SCT. Trends were seen for age, hemoglobin, and performance status at inclusion (Table 3). The OS curves for patients with the 1199A allele—heterozygous G/A—and the homozygous G/G genotype showed a similar pattern to TTP, but the difference between groups was not significant (Fig. 2a). Overall, there were no significant associations between the four *ABCB1* SNPs and either TTNT or OS.

**Table 3:** Cox regression analysis of TTP, forced entry method

Covariates	HR	95% CI	*P*
1199G>A SNP, G/A versus G/G genotype	0.280	0.074–1.054	0.060
Age	1.065	0.994–1.141	0.073
Gender (female compared to male)	0.693	0.297–1.621	0.398
Hemoglobin	1.031	0.999–1.063	0.059
Creatinine	1.009	0.996–1.021	0.184
Albumin	0.822	0.727–0.929	0.002
High- versus standard-risk cytogenetics (FISH)	3.890	1.719–8·805	0.001
Previous treatment^a^			
Thalidomide	1.640	0.512–5.250	0.405
Proteasome inhibitor + immunomodulatory drug	2.482	0.489–12.601	0.273
Other	0.518	0.203–1.320	0.168
ECOG performance status at inclusion^b^			
1	1.034	0.414–2.586	0.942
2	3.604	0.899–14.449	0.070
Not known	0.130	0·012–1.356	0.088
ISS disease stage at diagnosis^c^			
II	1.492	0.430–5.174	0.528
III	2.319	0.577–9.329	0.236
Not known	1.348	0.397–4.579	0.632
Previous versus no previous HDT-SCT	6.567	1.832–23.535	0.004

*CI* confidence interval, *ECOG* Eastern Cooperative Oncology Group, *FISH* fluorescence in situ hybridization, *HDT-SCT* high-dose therapy plus stem cell transplantation, *HR* hazard ratio, *ISS* International Staging System, *SNP* single-nucleotide polymorphism, *TTP* time to progression

^a^Compared with bortezomib treatment

^b^Compared with ECOG 0

^c^Compared with Stage I

Analysis of TTP according to 1199G>A genotype within patient subgroups defined by the presence or absence of high-risk cytogenetic features revealed that the influence of 1199G>A genotype on TTP appeared to be mainly restricted to patients with standard-risk cytogenetics (Fig. 1b); however, patient numbers were low (*n* = 40). Patients within the standard-risk subgroup did not differ between genotypes in terms of baseline factors (age, hemoglobin, albumin, creatinine, performance status, ISS stage, or previous treatment including HDT-SCT; *P* > 0.05, data not shown). Mean TTP was 2·3 years (95% CI 1.8–2.8) and 4.3 years (95% CI 3.7–4.9) for standard-risk patients with the G/G and G/A genotypes, respectively (*P* = 0.034); overall mean TTP in standard-risk patients was 2.8 years (95% CI 2.3–3.4). The OS curves for standard-risk patients showed a similar pattern to those for TTP, but the difference between groups was not significant (Fig. 2b). In addition, DoR appeared to be prolonged in standard-risk patients with the G/A genotype versus standard-risk patients carrying the G/G genotype [3.4 years (95% CI 1.43–2.45) versus 1.94 years (95% CI 2.88–3.87); *P* = 0.056]. No significant influence of 1199G>A genotype on TTP or OS was seen in the high-risk subgroup (Figs. 1c, 2c). Analyses carried out in the small subgroups of patients who were randomized to Len + Dex (*n* = 23) or single-agent Len (*n* = 24) in the interventional phase II trial found no significant differences in response parameters or survival times between *ABCB1* SNP genotypes (all *P* > 0.05, data not shown).

### Hematological AEs

Among the total population of 90 patients, grade 1–2 and grade 3–4 neutropenia were reported in 27 (30.0%) and 32 (35.6%) patients, respectively, grade 1–2 and grade 3–4 thrombocytopenia in 44 (48.9%) and six (6.7%) patients, respectively, and grade 1–2 and grade 3–4 anemia in 29 (32.2%) and four (4.4%) patients, respectively. No significant associations were found between the risk for, or severity of, hematological AEs and any of the SNPs investigated. Upper airway infection, fatigue, diarrhea, back pain and pneumonia were the most common non-hematological AEs (frequency > 10%) and, with the exception of six incidences of grade 3–4 pneumonia, all AEs were mild to moderate (supplemental Table SIV). However, frequencies of non-hematological AEs with suspected relation to the study drug were too low to make any associations with genotype.

## Discussion

In this study, we investigated the impact of four *ABCB1* SNPs on outcomes and AE frequency in 90 patients who received the second-line Len + Dex treatment for RRMM. *ABCB1*, which encodes the drug-transporting protein P-gp, is a polymorphic gene, and SNPs that potentially affect protein expression and function may also affect the subsequent outcome of treatment with P-gp substrates. As Len is excreted mainly via the kidneys and does not undergo extensive metabolism, variation in drug transporter function at the brush border of renal tubular cells was thought to be a potential contributor to differences in Len plasma concentrations, which could, hypothetically, affect the outcome of treatment. A number of studies have been published on the impact of *ABCB1* SNPs on outcomes with other treatment regimens in MM [6–9], however, to the best of our knowledge, no study has previously investigated associations between *ABCB1* SNPs and Len treatment outcomes.

No significant relationships between *ABCB1* genotype and best response, TTNT, OS, or the frequency of hematological AEs were demonstrated for any of the four SNPs. This is consistent with the findings of Schilthuizen et al., who also found no correlation with outcomes following induction chemotherapy or HDT-SCT for the 1236C>T, 2677G>T/A and 3435C>T polymorphisms [9]. In contrast, Drain et al. and Maggini et al. reported correlations of survival with both 3435C>T and 2677G>T/A genotype [7, 8]. Buda et al. demonstrated a trend towards improved TTP, progression-free survival, and response rates for patients with the 3435T variant when treated with doxorubicin and bortezomib, but not for patients treated with single-agent bortezomib [6]. Our results do not support any correlation with TTP or response rate for this SNP, which is in accordance with the result of the bortezomib arm in the study of Buda et al. However, the Kaplan–Meier curve showed a clear trend towards improved TTP, and there was a trend towards prolonged DoR, in patients carrying the 1199A variant allele versus patients with the homozygous G/G genotype. There was a difference in mean TTP of 1 year between the genotype subgroups, which is a rather dramatic and clinically relevant difference in relapsed patients with an incurable malignancy associated with high mortality rates. This trend was also clear in the multivariable Cox regression analysis of TTP, with a *P* value of 0.06. Subgroup analysis indicated that the influence of this genetic variant was limited mainly to patients with standard-risk cytogenetics, in whom the difference in mean TTP between genotype groups was 2 years. This is perhaps not unexpected, as it is likely that deleterious structural aberrations may overcome subtle variations in drug transporter activity. Nevertheless, in view of the small size of the subgroups in our analysis, this result requires confirmation in a larger cohort.

The 1199G>A SNP has not previously been investigated in patients with MM, but it has been studied in other malignancies and in in vitro settings. Although the effect of the 1199A variant appears to be substrate-specific, most previous studies reported either a decreased intracellular accumulation for the A variant or no differences between the alleles [16, 20–22]. One recent study in pediatric patients with acute lymphoblastic leukemia treated with protocols including methotrexate, prednisolone, doxorubicin, and vincristine demonstrated an increased risk of relapse for the heterozygous G/A genotype [23]. Similar findings have also been reported for patients with ovarian cancer treated with paclitaxel [24], and for patients with acute myeloid leukemia who received standard therapy including daunorubicin and cytarabine [19]. Based on these results, it would have been reasonable to expect that outcomes in our patient group would be worsened with the G/A genotype, owing to increased kidney excretion of Len; however, we found the opposite to be true. The findings in our analysis are consistent with those reported by Elens et al., who observed increased liver and blood concentrations of tacrolimus for the heterozygous G/A genotype, indicating a decreased transport activity of the variant allele [25].

In vivo studies have shown that co-administration of a P-gp inhibitor impacts the pharmacokinetics of Len [14, 26]. However, a recently published review argues that these studies on P-gp and Len suffered from problems such as the absence of controls and limited sample size, and that P-gp does not significantly impact Len pharmacokinetics In vivo [27]. This conclusion is based mainly on controlled trials of Len and known P-gp substrates and inhibitors in healthy volunteers, which showed no difference in Len pharmacokinetic parameters including systemic exposure and maximal plasma concentration [28].

It is well known that correlation does not necessarily mean causality, and the *ABCB1* 1199A variant may be linked to other genetic markers responsible for differences in outcomes. In addition, mechanisms of resistance other than drug efflux have been proposed and demonstrated for P-gp, including interaction with both intrinsic and extrinsic apoptosis pathways [29–31]. Interestingly, Glaski et al. demonstrated P-gp-dependent resistance to the extrinsic tumor necrosis factor-related apoptosis-inducing ligand (TRAIL) apoptosis signaling pathway in malignant cells [29]. The resistance to TRAIL-induced apoptosis required an active transporter and not just P-gp expression, indicating that the interaction was not on a transcriptional level. It was also shown that TRAIL was neither a substrate for P-gp nor an indirect modifier of P-gp conformation in the presence of known substrates. The authors speculated that cross-talk between P-gp and TRAIL receptors within their common membrane lipid raft microdomains may occur, but the exact mechanism for the interaction is yet to be fully elucidated. A functional role of this apoptosis signaling pathway for Len-mediated natural killer (NK)-cell activity towards MM cells has been demonstrated [32]. Considering these results, it might be proposed that altered P-gp function due to the 1199G>A SNP may influence Len-mediated NK-cell-dependent apoptosis through the TRAIL pathway, thus suggesting an alternative, drug efflux-independent explanation for the differences in treatment outcome seen in our patients.

In conclusion, we found no statistically significant influence of *ABCB1* genetic variants on Len treatment response, outcomes, or the risk of hematological AEs, indicating that these genotypes do not have a clinically relevant impact on the efficacy or safety of Len. However, the SNP 1199G>A displayed a clear trend towards an impact on TTP and DoR, and a similar pattern was observed in the OS data. Longer follow-up times could potentially yield clearer survival curve patterns; however, given our modest sample size and the relatively low frequency of the 1199A variant, the present results should be interpreted with caution and investigated further in a larger, well-characterized MM study population. Such a study would preferably include plasma and/or urine drug concentration measurements, in addition to further investigations of apoptosis signaling and markers in relation to *ABCB1* genotype. A genome-wide approach may also give more insight into the influence of gene variation on Len pharmacokinetics and treatment outcome, facilitating more tailored treatment of MM in the future.

## Supplemental material

Pharmacogenetic study of the impact of ABCB1 single nucleotide polymorphisms on lenalidomide treatment outcomes in patients with multiple myeloma: results from a phase IV observational study and subsequent phase II clinical trial
