# Supplementary material for: Pharmacogenetic study of the impact of ABCB1 single-nucleotide polymorphisms on lenalidomide treatment outcomes in patients with multiple myeloma: results from a phase IV observational study and subsequent phase II clinical trial
Source: Cancer Chemother Pharmacol. 2017 Nov 25;81(1):183–93. doi: 10.1007/s00280-017-3481-8 (PMC5754426; doi:10.1007/s00280-017-3481-8)
Supplement: Supplementary file 1 — Pharmacogenetic study of the impact of ABCB1 single nucleotide polymorphisms on lenalidomide treatment outcomes in patients with multiple myeloma: results from a phase IV observational study and subsequent phase II clinical trial [file 280_2017_3481_MOESM1_ESM.doc]

**Supplemental material**

**Pharmacogenetic study of the impact of ABCB1 single nucleotide polymorphisms on lenalidomide treatment outcomes in patients with multiple myeloma: results from a phase IV observational study and subsequent phase II clinical trial**

**Ingrid Jakobsen Falk^1^,*, Johan Lund^2^,*, Henrik Gréen^1,3^, Astrid Gruber^2^, Evren Alici^2^, Birgitta Lauri^4^, Cecilie Blimark^5^, Ulf-Henrik Mellqvist^6^, Agneta Swedin^7^, Karin Forsberg^8^, Conny Carlsson^9^, Mats Hardling^10^, Lucia Ahlberg^11^, Kourosh Lotfi^1,11^, and Hareth Nahi^2^**

*^1^Division of Drug Research, Department of Medical and Health Sciences, Linköping University, Linköping, Sweden, ^2^Unit for Hematology, Department of Medicine, Karolinska Institute, Huddinge, Sweden, ^3^Department of Forensic Genetics and Forensic Toxicology, National Board of Forensic Medicine, Linköping, Sweden, ^4^Department of Internal Medicine, Sunderby Hospital, Luleå, Sweden, ^5^Hematology Department, Sahlgrenska University Hospital, Gothenburg, Sweden, ^6^Department of Medicine, Division for Haematology, Oncology and Lung, South Elvsborg Hospital, Borås, Sweden, ^7^Hematology Department, Skåne University Hospital, Lund, Sweden, ^8^Department of Hematology, Norrland University Hospital, Umeå, Sweden, ^9^Department of Internal Medicine, Hallands Hospital, Halmstad, Sweden, ^10^Department of Hematology, Uddevalla Hospital, Uddevalla, Sweden and ^11^Department of Hematology, Linköping University Hospital, Linköping, Sweden.*

*IJF and JL share first authorship.

**Corresponding author:** Ingrid Jakobsen Falk, Division of Drug Research, Department of Medical and Health Sciences, Linköping University, Linköping, Sweden.

E-mail: [Ingrid.jakobsen.falk@liu.se](mailto:Ingrid.jakobsen.falk@liu.se). Tel: +46(0)10-1032029. ORCID 0000-0003-4450-0333.

**Table SI**. PCR and sequencing primers and nucleotide dispensing orders.

| ***ABCB1*** v**ariant** | **Sequence** |
| --- | --- |
| **1199G >A (Ser400Asn, rs2229109)** |  |
| **PCR primer, forward biotinylated**  **PCR primer, reverse**  **Sequencing primer**  **Nucleotide dispensing order** | ATTGACAGCTATTCGAAGAGTG  CCTTAACTTCTTTTCGAGATGG  CTTTTCGAGATGGGTAA  GACTCGAGTG |
| **1236C>T (silent, rs1128503)** |  |
| **PCR primer, forward biotinylated**  **PCR reverse**  **Sequencing primer**  **Nucleotide dispensing order** | GTCTGTGAATTGCCTTGAAGT  CAGCCACTGTTTCCAACC  TGCACCTTCAGGTTCA  TGAGCTCAG |
| **2677G>T/A (Ala893Ser, rs2032582)** |  |
| **PCR primer, forward biotinylated**  **PCR primer, reverse**  **Sequencing primer**  **Nucleotide dispensing order** | GGACAAGCACTGAAAGATAAG  AGGGAGTAACAAAATAACACTGAT  TTAGTTTGACTCACCTTCC  GCAGTCAGCT |
| **3435C>T (silent, rs1045642)** |  |
| **PCR primer, forward biotinylated**  **PCR primer, reverse**  **Sequencing primer**  **Nucleotide dispensing order** | GCAAAGAAATAAAGCGACTGAA  TTGAAGAGAGACTTACATTAGGCAG  GTGGTGTCACAGGAAGA  CGATCAGTG |

PCR, polymerase chain reaction.

**Table SII.** Distribution of patient characteristics between *ABCB1* genotypes. Age, hemoglobin, albumin and creatinine distributions (presented below with mean values and range) were compared using Mann-Whitney U or Kruskal-Wallis tests, and categorical variables were investigated with chi-square or generalized Fisher´s exact tests. Significant *P*-values (≤0.05) are indicated in bold. Low frequency 2677A allele excluded from the analysis (n=3).

| **Variable** | ***ABCB1* genotype** | | | | ***P*** |
| --- | --- | --- | --- | --- | --- |
|  | **1199G>A, rs2229109** | | | |  |
|  | G/G | | G/A | |  |
| Age, years | 67·2 (42·.1-85·9) | | 66·3 (52·6-78·8) | | 0·64 |
| Gender;  Female  Male | 34  42 | | 7  7 | | 0·78 |
| Hemoglobin, g/L (range) | 116 (63-155) | | 110 (87-131) | | 0·486 |
| Albumin, g/L (range) | 35·1 (25-44) | | 36 (30-39) | | 0·495 |
| Creatinine, μmol/L (range) | 82·1 (43-270) | | 77·7 (42-111) | | 0·885 |
| Risk assessed by FISH;  High  Standard-risk | 32  33 | | 3  7 | | 0·32 |
| Previous HDT-SCT (N=90);  Yes  No | 41  35 | | 9  5 | | 0·57 |
| Previous treatment;  Velcade  Thalidomide  PI + IMiD  Other | 45  9  4  18 | | 6  1  2  5 | | 0·33 |
| ECOG performance status at inclusion;  0  1  2  Not known | 32  34  7  3 | | 7  5  0  2 | | 0·30 |
| ISS stage at inclusion  1  2  3  Not known | 13  37  8  18 | | 3  4  2  5 | | 0·47 |
|  | **1236C>T, rs1128503** | | | |  |
|  | **C/C** | **C/T** | | **T/T** |  |
| Age, years | 70·4 (49·1-85·9) | 64·7 (42·1-81·5) | | 64·4 (44·9-78·1) | **0**·**018** |
| Gender;  Female  Male | 19  19 | 16  23 | | 6  7 | 0·74 |
| Hemoglobin, g/L (range) | 113 (89·148) | 115 (63-147) | | 116 (86-144) | 0·70 |
| Albumin, g/L (range) | 35·6 (25-42) | 34·3 (25-42) | | 35·9 (28-43) | 0·36 |
| Creatinine, μmol/L (range) | 83·1 (42-148) | 81·9 (48-270) | | 76·5 (50-158) | 0·64 |
| Risk assessed by FISH;  High  Standard-risk | 16  16 | 16  18 | | 3  6 | 0·76 |
| Previous HDT-SCT;  Yes  No | 15  23 | 27  12 | | 8  5 | **0**·**028** |
| Previous treatment;  Velcade  Thalidomide  PI + IMiD  Other | 22  5  2  9 | 21  4  3  11 | | 8  1  1  3 | 1·0 |
| ECOG performance status at inclusion;  0  1  2  Not known | 15  17  4  2 | 17  18  2  2 | | 7  4  1  1 | 0·91 |
| ISS stage at inclusion  1  2  3  Not known | 9  18  3  8 | 5  19  6  9 | | 2  4  1  6 | 0·51 |
|  | **2677G>T, rs2032582** | | | |  |
|  | **G/G** | **G/T** | | **T/T** |  |
| Age, years | 71·0 (46·5-85·9) | 64·9 (42·1-81·5) | | 64·4 (44·9-78·1) | **0**·**008** |
| Gender;  Female  Male | 18  17 | 16  23 | | 6  7 | 0·74 |
| Hemoglobin, g/L (range) | 115 (89-148) | 113 (63-147) | | 116 (86-144) | 0·92 |
| Albumin, g/L (range) | 35·4 (25-42) | 34·5 (25-42) | | 35·9 (28-43) | 0·64 |
| Creatinine, μmol/L (range) | 82·4 (42-148) | 83·5 (43-270) | | 76·5 (50-158) | 0·68 |
| Risk assessed by FISH;  High  Standard-risk | 15  14 | 17  17 | | 3  6 | 0·68 |
| Previous HDT-SCT;  Yes  No | 11  24 | 28  11 | | 8  5 | **0**·**002** |
| Previous treatment;  Velcade  Thalidomide  PI + IMiD  Other | 22  5  2  6 | 19  4  3  13 | | 8  1  1  3 | 0·77 |
| ECOG performance status at inclusion;  0  1  2  Not known | 15  15  3  2 | 17  18  2  2 | | 7  4  1  1 | 0·95 |
| ISS stage at inclusion  1  2  3  Not known | 8  17  3  7 | 5  18  6  10 | | 2  4  1  6 | 0·57 |
|  | **3435C>T, rs1045642** | | | |  |
|  | **C/C** | **C/T** | | **T/T** |  |
| Age, years | 70·8 (49·1-82·8) | 67·3 (42·1-85·9) | | 63·5 (44·9-81·5) | **0**·**046** |
| Gender;  Female  Male | 10  8 | 21  28 | | 10  13 | 0·66 |
| Hemoglobin, g/L (range) | 113 (89-148) | 114 (63-148) | | 116 (86-144) | 0·83 |
| Albumin, g/L (range) | 35·6 (25-42) | 34·6 (25-42) | | 35·6 (26-43) | 0·32 |
| Creatinine, μmol/L (range) | 86·5 (52-148) | 83·3 (42-270) | | 74·4 (43-158) | 0·39 |
| Risk assessed by FISH;  High  Standard-risk | 6  9 | 21  22 | | 8  9 | 0·90 |
| Previous HDT-SCT;  Yes  No | 5  13 | 32  17 | | 13  10 | **0**·**024** |
| Previous treatment;  Velcade  Thalidomide  PI + IMiD  Other | 10  4  0  4 | 26  5  5  13 | | 15  1  1  6 | 0·57 |
| ECOG performance status at inclusion;  0  1  2  Not known | 6  7  4  1 | 23  23  1  2 | | 10  9  2  2 | 0·19 |
| ISS stage at inclusion  1  2  3  Not known | 5  10  1  2 | 8  22  7  12 | | 3  9  2  9 | 0·46 |

ECOG, Eastern Cooperative Oncology Group; FISH, fluorescence in situ hybridization; HDT-SCT, high-dose therapy plus stem cell transplantation; IMiD, immunomodulatory drug; ISS, International Staging System; PI, proteasome inhibitor.

**Table SIII.** Response rates according to *ABCB1* SNP genotypes. The minor allele 2677A (n=3) was excluded from the analysis. Chi-square or generalized Fisher´s exact tests, with significant *P*-values (≤0.05) indicated in bold. Low frequency 2677A allele excluded from the analysis (n=3).

| **Response** | **ABCB1 SNP** | | | | | | | **Chi-square distributions** |
| --- | --- | --- | --- | --- | --- | --- | --- | --- |
|  | **1199G>A** | | | | | | |  |
| **First response** | G/G, *n* = 76 (84·4%) | | | G/A, *n* = 14 (15·6%) | | | |  |
| **CR**  **nCR**  **VGPR**  **PR**  **Minimal/no response**  **Progression**  **≥PR**  **≥VGPR** | 1 (1·3%)  4 (5·3%)  4 (5·3%)  55 (72·4%)  10 (11·1%)  2 (2·6%)  64 (84·2%)  9 (11·8%) | | | 0  0  1 (7·1%)  12 (85·7%)  1 (7·1%)  0  13 (92·9%)  1 (7·1%) | | | | ≥PR vs <PR, *P* = 0·68  ≥VGPR vs <VGPR, *P* = 1·0 |
| **Best response** | G/G, *n* = 76 (84·4%) | | | G/A, *n* = 14 (15·6%) | | | |  |
| **CR**  **nCR**  **VGPR**  **PR**  **Minimal/no response**  **Progression**  **≥PR**  **≥VGPR** | 9 (11·8%)  12 (15·8%)  14 (18·4%)  29 (38·2%)  10 (13·2%)  2 (2·6%)  64 (84·2%)  35 (46·1%) | | | 3 (21·4%)  1 (7·1%)  3 (21·4%)  6 (42·9%)  1 (7·1%)  0  13 (92·9%)  7 (50·0%) | | | | ≥PR vs <PR *P* = 0·68  ≥VGPR vs <VGPR *P* = 1·0 |
|  | **1236C>T** | | | | | | |  |
| **First response** | C/C, *n* = 38 (42·2%) | | C/T, *n* = 39 (43·3%) | | | | T/T, *n* = 13 (14·4%) |  |
| **CR**  **nCR**  **VGPR**  **PR**  **Minimal/no response**  **Progression**  **≥PR**  **≥VGPR** | 0  1 (2·6%)  2 (5·3%)  29 (76·3%)  5 (13·2%)  1 (2·6%)  32 (84·2%)  3 (7·9%) | | 1 (2·6%)  1 (2·6%)  1 (2·6%)  31 (79·5%)  5 (12·8%)  0  34 (87·2%)  3 (7·7%) | | | | 0  2 (15·4%)  2 (15·4%)  7 (53·8%)  1(7·7%)  1 (7·7%)  11 (84·6%)  3 (23·1%) | ≥PR vs <PR,  *P* = 0·92  ≥VGPR vs <VGPR,  *P* = 0·091 |
| **Best response** | C/C, *n* = 38 (42·2%) | | C/T, *n* = 39 (43·3%) | | | | T/T, *n* = 13 (14·4%) |  |
| **CR**  **nCR**  **VGPR**  **PR**  **Minimal/no response**  **Progression**  **≥PR**  **≥VGPR** | 2 (5·3%)  6 (15·8%)  7 (18·4%)  17 (44·7%)  5 (13·2%)  1 (2·6%)  32 (84·2%)  15 (39·5%) | | 6 (15·4%)  4 (10·3%)  9 (23·1%)  15 (38·5%)  5 (12·8%)  0  34 (87·2%)  19 (48·7%) | | | | 4 (30·8%)  3 (23·1%)  1 (7·7%)  3 (23·7%)  1 (7·7%)  1 (7·7%)  11 (84·6%)  8 (61·5%) | ≥PR vs <PR, *P* = 0·92  ≥VGPR vs <VGPR,  *P* = 0·36 |
|  | **2677G>T** | | | | | | |  |
| **First response** | G/G, *n* = 35 (40·2%) | G/T, *n* = 39 (44·8%) | | | | T/T, *n* = 13 (14·9%) | |  |
| **CR**  **nCR**  **VGPR**  **PR**  **Minimal/no response**  **Progression**  **≥PR**  **≥VGPR** | 0  1 (2·9%)  1 (2·9%)  28 (80·0%)  4 (11·4%)  1 (2·9%)  30 (85·7%)  2 (5·7%) | 1 (2·6%)  0  1 (2·6%)  31 (79·5%)  6 (15·4%)  0  33 (84·6%)  2 (5·1%) | | | | 0  2 (15·4%)  2 (15·4%)  7 (53·8%)  1 (7·7%)  1 (7·7%)  11 (84·6%)  4 (30·8%) | | ≥PR vs <PR, *P* = 1·0  ≥VGPR vs <VGPR, ***P* = 0·037** |
| **Best response** | G/G, *n* = 35 (40·2%) | G/T, *n* = 39 (44·8%) | | | | T/T, *n* = 13 (14·9%) | |  |
| **CR**  **nCR**  **VGPR**  **PR**  **Minimal/no response**  **Progression**  **≥PR**  **≥VGPR** | 1 (2·9%)  5 (14·3%)  6 (17·1%)  18 (51·4%)  4 (11·4%)  1 (2·9%)  30 (85·7%)  12 (34·3%) | 6 (15·4%)  4 (10·3%)  9 (23·1%)  14 (35·9%)  6 (15·4%)  0  33 (84·6%)  19 (48·7%) | | | | 4 (30·8%)  3 (23·1%)  1 (7·7%)  3 (23·1%)  1 (7·7%)  1 (7·7%)  11 (84·6%)  8 (61·5%) | | ≥PR vs <PR, *P* = 1·0  ≥VGPR vs <VGPR, *P* = 0·20 |
|  | **3435C>T** | | | | | | |  |
| **First response** | C/C, *n* = 18 (20%) | C/T, *n* = 49 (54·4%) | | | T/T, *n* = 23 (25·6%) | | |  |
| **CR**  **nCR**  **VGPR**  **PR**  **Minimal/no response**  **Progression**  **≥PR**  **≥VGPR** | 0  1 (5·6%)  0  15 (83·3%)  1 (5·6%)  1 (5·6%)  16 (88·9%)  1 (5·6%) | 1 (2·0%)  0  2 (4·1%)  39 (79·6%)  7 (14·3%)  0  42 (85·7%)  3 (6·1%) | | | 0  3 (13·0%)  3 (13·0%)  13 (56·5%)  3 (3·0%)  1 (4·3%)  19 (82·6%)  6 (26·3%) | | | ≥PR vs <PR, *P* = 0·85  ≥VGPR vs <VGPR, ***P* = 0·04** |
| **Best response** | C/C, *n* = 18 (20%) | C/T, *n* = 49 (54·4%) | | | T/T, *n* = 23 (25·6%) | | |  |
| **CR**  **nCR**  **VGPR**  **PR**  **Minimal/no response**  **Progression**  **≥PR**  **≥VGPR** | 1 (5·6%)  1 (5·6%)  7 (38·9%)  7 (39%)  1 (5·6%)  1 (5·6%)  16 (88·9%)  9 (50·0%) | 7 (14·3%)  7 (14·3%)  7 (14·3%)  21 (42·9%)  7 (14·3%)  0  42 (85·7%)  21 (42·9%) | | | 4 (17·4%)  5 (21·7%)  3 (13·0%)  7 (30·4%)  3 (13·0%)  1 (4·3%)  19 (82·6%)  12 (52·2%) | | | ≥PR vs <PR, *P* = 0·85  ≥VGPR vs <VGPR,  *P* = 0·78 |

CR, complete response; nCR, near complete response; PR, partial response; SNP, single nucleotide polymorphism; VGPR, very good partial response.

**Table SIV. Non-hematological adverse events reported in >10% of 90 patients.**

| **Adverse event** | **All, *N* (%)** | **Grade 3–4, *N* (%)** | **Relation to study drug, *n*/*N* reported (%)** |
| --- | --- | --- | --- |
| **Upper airway infection** | 23 (25·6) | 0 | 3/23 (13) |
| **Fatigue** | 16 (17·8) | 0 | 14/16 (87·5) |
| **Diarrhea** | 16 (17·8) | 0 | 4/16 (25) |
| **Pneumonia** | 12 (13·3) | 6 (6·7) | 5/12 (41·7) |
| **Back pain** | 12 (13·3) | 0 | 0 |
